# PRMT5 Is Involved in Spermatogonial Stem Cells Maintenance by Regulating *Plzf* Expression via Modulation of Lysine Histone Modifications

**DOI:** 10.3389/fcell.2021.673258

**Published:** 2021-05-21

**Authors:** Fangfang Dong, Min Chen, Min Chen, Lin Jiang, Zhiming Shen, Longfei Ma, Chunsheng Han, Xudong Guo, Fei Gao

**Affiliations:** ^1^State Key Laboratory of Stem Cell and Reproductive Biology, Institute of Zoology, Chinese Academy of Sciences, Beijing, China; ^2^University of Chinese Academy of Sciences, Beijing, China; ^3^Institute for Stem Cell and Regeneration, Chinese Academy of Sciences, Beijing, China; ^4^Guangdong and Shenzhen Key Laboratory of Male Reproductive Medicine and Genetics, Institute of Urology, Peking University Shenzhen Hospital, Shenzhen Peking University-Hong Kong University of Science and Technology Medical Center, Shenzhen, China; ^5^School of Basic Medical Sciences, Zunyi Medical University, Zunyi, China; ^6^State Key Laboratory of Reproductive Regulation and Breeding of Grassland Livestock, Inner Mongolia University, Hohhot, China

**Keywords:** PRMT5, spermatogonial stem cells, histone lysine modification, lysine demethylase, PLZF

## Abstract

Protein arginine methyltransferase 5 (PRMT5) catalyzes the formation of mono- or symmetric dimethylarginine residues on histones and non-histone substrates and has been demonstrated to play important roles in many biological processes. In the present study, we observed that PRMT5 is abundantly expressed in spermatogonial stem cells (SSCs) and that *Prmt5* deletion results in a progressive loss of SSCs and male infertility. The proliferation of *Prmt5*-deficient SSCs cultured *in vitro* exhibited abnormal proliferation, cell cycle arrest in G0/G1 phase and a significant increase in apoptosis. Furthermore, PLZF expression was dramatically reduced in *Prmt5*-deficient SSCs, and the levels of H3K9me2 and H3K27me2 were increased in the proximal promoter region of the *Plzf* gene in *Prmt5*-deficient SSCs. Further study revealed that the expression of lysine demethylases (JMJD1A, JMJD1B, JMJD1C, and KDM6B) was significantly reduced in *Prmt5*-deficient SSCs and that the level of permissive arginine methylation H3R2me2s was significantly decreased at the upstream promoter region of these genes in *Prmt5*-deficient SSCs. Our results demonstrate that PRMT5 regulates spermatogonial stem cell development by modulating histone H3 lysine modifications.

## Introduction

Spermatogenesis is a highly precise cellular process, which consists of the self-renewal and differentiation of spermatogonial stem cells, spermatocyte meiosis and post-meiotic development of spermatids (He et al., 2008; Wu et al., 2016). Spermatogonia contain a small population of germline-specific stem cells with the ability to self-renew and differentiation. The differentiation of spermatogonia is stimulated by both intrinsic and extrinsic factors, which subsequently generate differentiating spermatogonia, spermatocytes, spermatids, and mature sperm (Chen and Liu, 2015; Mäkelä and Hobbs, 2019). In mammals, both genetic and epigenetic modifications are involved in the development of male germ cells. *Plzf*, *Gfra1*, *Pou5f1*, *Lin28A*, and *Nanos3* are expressed in undifferentiated spermatogonia, and *c-Kit* is considered a marker gene of differentiating spermatogonia. Several Sertoli cell-produced growth factors, such as glial cell line-derived neurotrophic factor (GDNF), fibroblast growth factor (FGF2), and insulin-like growth factor (IGF), are also crucial for the maintenance and proliferation of spermatogonial stem cells (SSCs) (Oatley and Brinster, 2008; Zhang et al., 2012; Wang et al., 2016; Chen et al., 2017). Homozygous deletion of *Gdnf* leads to major defects in neonatal death. The function of *Gdnf* in SSC maintenance was first discovered in *Gdnf* heterozygous mutant (Meng et al., 2000).

*Plzf* (also known as *Zfp145*, *Zbtb16*, and promyelocytic leukemia zinc-finger) belongs to the Kruppel family and is expressed in hematopoietic stem/progenitor cells (Reid et al., 1995; Liu et al., 2016; Hai et al., 2019; Poplineau et al., 2019), bone marrow progenitor cells (Shaknovich et al., 1998), mesenchymal stem cells (Agrawal Singh et al., 2019) and other somatic cells (Cook et al., 1995; Barna et al., 2000). In mammalian testes, PLZF was first detected in prospermatogonia at E17.5 and is continually expressed in SSCs and spermatogonial progenitor cells (SPCs) at the adult stage (Avantaggiato et al., 1995; Buaas et al., 2004; Costoya et al., 2004). PLZF has been reported to play an essential role in SSCs pool maintenance and in regulating the self-renewal of SSCs. *Plzf* knockout leads to progressive germ cell loss after birth, and the spermatogonia are visible in only a few seminiferous tubules of adult mice (Buaas et al., 2004; Costoya et al., 2004).

PRMT5, a type II protein arginine methyltransferase, catalyzes the transfer of a methyl group from S-adenosylmethionine (SAM or AdoMet) to histones and non-histone substrates to form mono- or symmetric dimethylarginine (MMA or sDMA, respectively) (Rho et al., 2001; Bedford, 2007; Stopa et al., 2015). PRMT5 plays important roles in diverse cellular processes, such as cell differentiation, cell cycle, apoptosis, tumorigenesis and spliceosome assembly (Pahlich et al., 2006; Stopa et al., 2015; Hamard et al., 2018; Raposo and Piller, 2018). Previous studies have demonstrated that PRMT5 is required for germ cell development, as the loss of *Prmt5* in primordial germ cells (PGCs) causes male and female sterility. PRMT5 represses the activation of LINEs and IAP transposons via symmetric dimethylation of arginine 3 on histone H2A and H4 (H2A/H4R3me2s) (Kim et al., 2014). PRMT5 is also required for PGC survival by promoting methylation of Sm spliceosomal proteins (Li et al., 2015). The results of our previous study demonstrated that inactivation of *Prmt5* in male germ cells using *Stra8-Cre* causes aberrant spermatogenesis and male infertility (Wang et al., 2015c), suggesting that PRMT5 is essential for the development of male germ cells. PRMT5 is also expressed in mouse spermatogonial stem cells (SSCs). However, whether PRMT5 is involved in the development of SSCs is unknown. In the present study, we demonstrated that the deletion of *Prmt5* in germ cells resulted in loss of spermatogonial stem cells (SSCs) and male infertility. *Prmt5*-deficient SSCs cultured *in vitro* exhibited abnormal proliferation, and the cell cycle was arrested in G0/G1 phase. Further study revealed that the expression of PLZF was dramatically reduced in *Prmt5*-deficient SSCs. We also observed that inactivation of *Prmt5* resulted in enrichment of H3K9me2 and H3K27me2 in the promoter region of the *Plzf* gene, which in turn caused downregulation of *Plzf* expression and defects in SSC development.

## Results

### Deletion of *Prmt5* in Spermatogonia Causes Germ Cell Loss and Male Infertility

PRMT5 was previously reported to be expressed in PGCs during the embryonic stage (Ancelin et al., 2006; Kim et al., 2014; Li et al., 2015) and in spermatocytes postnatally (Wang et al., 2015c; Dong et al., 2019). In the present study, PRMT5 expression in spermatogonia was examined by immunofluorescence and western blot assays. As shown in Supplementary Figure 1, PRMT5 was primarily detected in the cytoplasm of PLZF-positive germ cells (Supplementary Figure 1C, white arrowheads). PRMT5 was also detected in both the nucleus and cytoplasm of SSCs cultured *in vitro* by both immunofluorescence (Supplementary Figure 1D, white arrows) and western blot analysis. Lamin a/c and GAPDH served as loading controls for the nuclear and cytoplasmic extracts, respectively (Supplementary Figure 1G).

**FIGURE 1:**
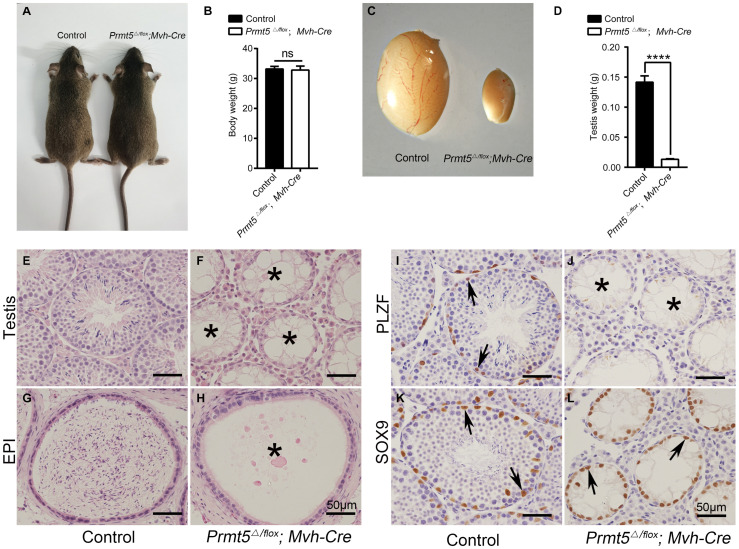
Deletion of *Prmt5* causes germ cell loss and male infertility. No obvious developmental abnormalities were observed in adult *Prmt5^Δ/flox^;Mvh-Cre* mice **(A)**, and the body weights were comparable to those of control littermates **(B)**. The size of testes from *Prmt5^Δ/flox^;Mvh-Cre* mice was significantly smaller than that of control littermates **(C,D)**. Germ cells at different developmental stages were observed in control testes **(E)**, whereas most seminiferous tubules were empty in adult *Prmt5^Δ/flox^;Mvh-Cre* testes **(F**, asterisks). The cauda epididymis of control males was filled with mature sperm **(G)**, but no mature sperm were observed in the epididymis of *Prmt5*-deficient males **(H)**. PLZF-positive spermatogonia were located at the peripheral region of the seminiferous tubules in control mice [**(I)**, black arrows], whereas no PLZF-positive spermatogonia were observed in *Prmt5^Δ/flox^;Mvh-Cre* mice **(J)**. SOX9-positive Sertoli cells were observed in the seminiferous tubules of both control [**(K)**, black arrows] and *Prmt5^Δ/flox^;Mvh-Cre* mice [**(L)**, black arrows]. Error bars represent the SEM of triplicate results. ****P < 0.00001 indicates a significant difference (*t*-test).

To assess the functions of PRMT5 in the development of SSCs, *Prmt5* was deleted in prospermatogonia from E15 by crossing with *Mvh-Cre* transgenic mice (Gallardo et al., 2007). The immunofluorescence showed that PRMT5 was completely deleted in the PLZF-positive germ cells of *Prmt5^Δ/flox^;Mvh-Cre* testes (Supplementary Figures 2D–F, white arrows). No obvious developmental abnormalities were observed in adult *Prmt5^Δ/flox^;Mvh-Cre* mice (Figure 1A), and their body weights were comparable to those of control littermates (Figure 1B). However, the size of testes from *Prmt5^Δ/flox^;Mvh-Cre* mice was significantly reduced (Figures 1C,D). The histological results showed that germ cells at different developmental stages were observed in control testes (Figure 1E), whereas most of the seminiferous tubules in adult *Prmt5^Δ/flox^;Mvh-Cre* testes lacked germ cells (Figure 1F, asterisks). The cauda epididymis of control males was filled with mature sperm (Figure 1G), but no mature sperm were observed in *Prmt5*-deficient males (Figure 1H, asterisks). PLZF-positive spermatogonia were located at the peripheral region of the seminiferous tubules in control mice (Figure 1I, black arrows), whereas no PLZF-positive spermatogonia were observed in *Prmt5^Δ/flox^;Mvh-Cre* mice (Figure 1J, asterisks). SOX9-positive Sertoli cells were observed in the seminiferous tubules of both control (Figure 1K, black arrows) and *Prmt5^Δ/flox^;Mvh-Cre* mice (Figure 1L, black arrows). These results indicate that PRMT5 is important for the survival and maintenance of spermatogonial stem cells.

### The Germ Cells in *Prmt5^Δ/flox^;Mvh-Cre* Mice Were Gradually Lost From P10

**FIGURE 2:**
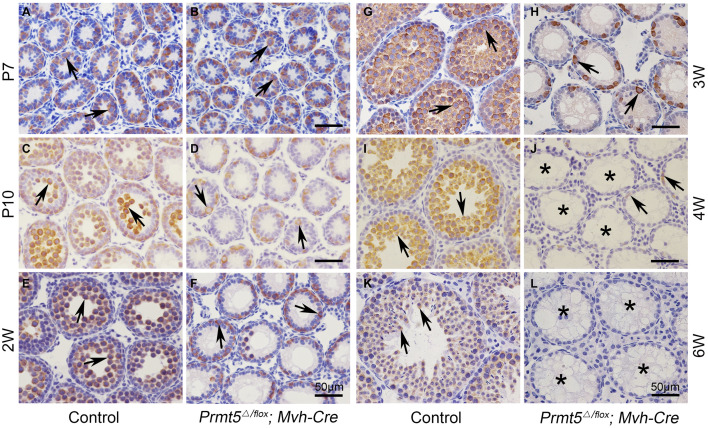
Germ cells from *Prmt5^Δ/flox^;Mvh-Cre* mice are gradually lost from P10. The germ cells in control and *Prmt5^Δ/flox^;Mvh-Cre* mice were labeled with an anti-MVH antibody. MVH-positive germ cells were observed in both control [**(A)**, black arrows] and *Prmt5^Δ/flox^;Mvh-Cre* mice [**(B)**, black arrows] at P7, and no difference was noted. The number of germ cells in *Prmt5^Δ/flox^;Mvh-Cre* mice [**(D)**, black arrows] was significantly reduced at P10 compared to that observed in control mice [**(C)**, black arrows]. The number of germ cells was significantly increased from 2 to 6 weeks in control mice [**(E,G,I,K)**, black arrows]. In contrast, the number of germ cells in *Prmt5^Δ/flox^;Mvh-Cre* mice was gradually reduced from 2 to 4 weeks [**(F,H,J)**, black arrows] and was completely absent at 6 weeks of age [**(L)**, asterisks].

Testes from *Prmt5^Δ/flox^;Mvh-Cre* mice (Supplementary Figure 3B) were grossly normal at P7 compared to those of control testes (Supplementary Figure 3A). Aberrant seminiferous tubules were first noted in *Prmt5^Δ/flox^;Mvh-Cre* testes at P10 (Supplementary Figure 3D, asterisks). Empty tubules were observed in *Prmt5*-deficient testes at 2 weeks (Supplementary Figure 3F, asterisks), 3 weeks (Supplementary Figure 3H, asterisks), 4 weeks (Supplementary Figure 3J, asterisks) and 6 weeks (Supplementary Figure 3L, asterisks). The development of germ cells at different developmental stages was also examined by immunohistochemistry. MVH-positive germ cells were observed in both control (Figure 2A, black arrows) and *Prmt5^Δ/flox^;Mvh-Cre* mice (Figure 2B, black arrows) at P7, and no difference was noted. The number of germ cells in *Prmt5^Δ/flox^;Mvh-Cre* mice (Figure 2D, black arrows) was significantly reduced at P10 than that in control mice (Figure 2C, black arrows), while the number of germ cells was significantly increased from 2 to 6 weeks in these mice (Figures 2E,G,I,K, black arrows). In contrast, the number of germ cells in *Prmt5^Δ/flox^;Mvh-Cre* mice was gradually reduced from 2 to 4 weeks (Figures 2F,H,J, black arrows), and they were completely absent at 6 weeks of age (Figure 2L, asterisks). PLZF-positive spermatogonia (green) were observed in the seminiferous tubules of control mice at 3 weeks (Supplementary Figure 4A, white arrows), 4 weeks (Supplementary Figure 4B, white arrows), and 6 weeks (Supplementary Figure 4C, white arrows). PLZF-positive spermatogonia were also observed in the seminiferous tubules of *Prmt5^Δ/flox^;Mvh-Cre* mice at 3 weeks (Supplementary Figure 4D, white arrows) and 4 weeks (Supplementary Figure 4E, white arrows) but not at 6 weeks (Supplementary Figure 4F, asterisks). The quantitative results showed that the number of PLZF-positive germ cells was dramatically reduced in *Prmt5^Δ/flox^;Mvh-Cre* mice at 3 weeks (Supplementary Figure 4G) and 4 weeks (Supplementary Figure 4H), and no germ cells were counted at 6 weeks (Supplementary Figure 4I). These results indicate that PRMT5 is required for the maintenance of the SSC pool.

### *Prmt5*-Deficient Germ Cells Were Defective for Meiosis

**FIGURE 3:**
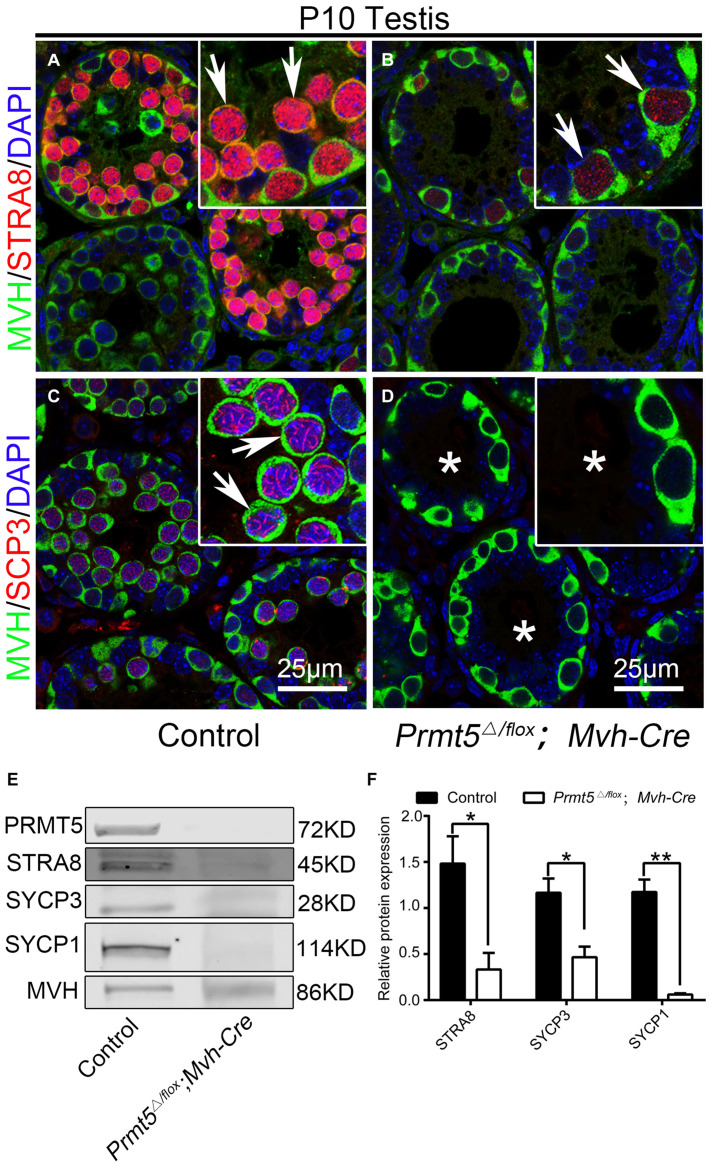
*Prmt5*-deficient germ cells exhibit a defect in meiosis initiation. A strong STRA8 signal was detected in the germ cells of control testes [**(A)**, white arrows], whereas only a weak STRA8 signal was detected in the germ cells of *Prmt5^Δ/flox^;Mvh-Cre* testes [**(B)**, white arrows]. SYCP3-positive germ cells were observed in control testes [**(C)**, white arrows], but no SYCP3 signal was detected in the germ cells of *Prmt5^Δ/flox^;Mvh-Cre* testes [**(D)**, asterisks]. The expression of meiosis-associated genes (STRA8, SYCP3 and SYCP1) was significantly reduced in *Prmt5^Δ/flox^;Mvh-Cre* testes at P10 **(E,F)**, The total protein detected from control testes was 10 μg, and the total protein detected from *Prmt5^Δ/flox^;Mvh-Cre* testes was 40 μg. MVH was used as a loading control in **(E)**, protein values were normalized to MVH and expressed as the mean ± SEM (*n* = 3), *P < 0.01, **P < 0.001 indicates a significant difference (*t*-test).

To test whether the process of meiosis is affected after *Prmt5* depletion, the expression of STRA8, SYCP3 and γH2AX at P10 was analyzed by immunofluorescence and western blot assays. A strong STRA8 signal was detected in the germ cells of control testes at P10 (Figure 3A, white arrows), whereas only very weak STRA8 expression was observed in the germ cells of *Prmt5^Δ/flox^;Mvh-Cre* testes (Figure 3B, white arrows). SYCP3-positive germ cells were observed in control testes (Figure 3C, white arrows), but no SYCP3 signal was detected in the germ cells of *Prmt5^Δ/flox^;Mvh-Cre* testes at P10 (Figure 3D, white asterisks). The western blot results showed that the expression of the meiosis-associated proteins STRA8, SYCP3 and SYCP1 was significantly reduced in *Prmt5^Δ/flox^; Mvh-Cre* testes at P10 (Figures 3E,F). These results indicate that meiosis is blocked in *Prmt5*-deficient germ cells.

### *Prmt5*-Deficient Spermatogonial Stem Cells Exhibited Abnormal Proliferation

**FIGURE 4:**
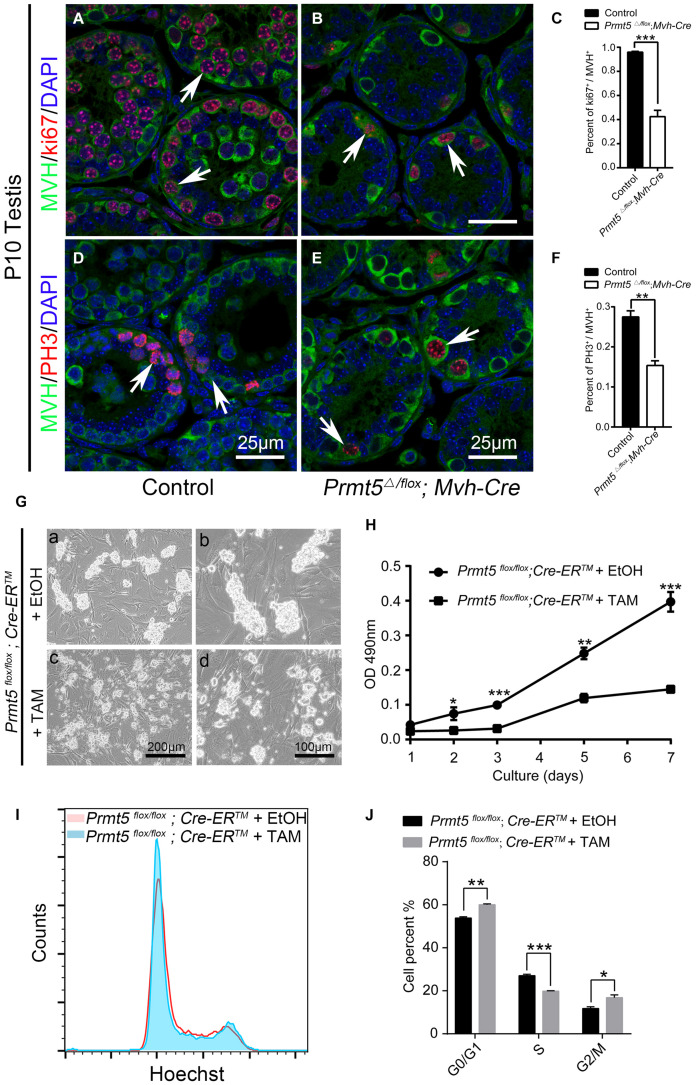
*Prmt5-*deficient spermatogonial stem cells exhibit abnormal proliferation. Ki67-positive (red) germ cells were detected in both control [**(A)**, white arrows] and *Prmt5^Δ/flox^;Mvh-Cre* testes [**(B)**, white arrows] at P10. The percentage of Ki67-positive germ cells was dramatically reduced in *Prmt5^Δ/flox^;Mvh-Cre* mice **(C)**. PH3 (red) was detected in MVH-positive germ cells of both control [**(D)**, white arrows] and *Prmt5^Δ/flox^;Mvh-Cre* testes [**(E)**, white arrows] at P10. The percentage of PH3-positive germ cells was dramatically reduced in *Prmt5^Δ/flox^;Mvh-Cre* mice **(F)**. **(G)** Bright-field images of control and *Prmt5-*deficient SSCs cultured *in vitro. Prmt5^*flox/flox*^;Cre-ER^TM^* SSCs were treated with ethanol or 1 μM tamoxifen to induce *Cre* activation. **(H)** MTT assay of cultured control and *Prmt5-*deficient SSCs. **(I)** FACS analysis of cultured control and *Prmt5-*deficient SSCs. **(J)** Quantitative analysis of cells in G0/G1, S, and G2 phase. Error bars represent the SEM of triplicate results. *P < 0.05, **P < 0.005, ***P < 0.0005 indicates a significant difference (*t*-test).

In the present study, we observed that PLZF-positive spermatogonial stem cells were gradually lost in *Prmt5* knockout mice. To test whether PRMT5 is involved in the proliferation or self-renewal of SSCs, germ cells were labeled with Ki67 and PH3. Ki67 and PH3 were detected in MVH-positive germ cells in both control (Figures 4A,D, white arrows) and *Prmt5^Δ/flox^;Mvh-Cre* testes (Figures 4B,E, white arrows) at P10. The quantitative results showed that the percentage of Ki67- and PH3-positive germ cells was dramatically reduced in *Prmt5^Δ/flox^;Mvh-Cre* mice (Figures 4C,F). These results indicate that the deletion of *Prmt5* leads to defects in germ cell proliferation. To further confirm these results, SSCs from *Prmt5^*flox/flox*^;Cre-ER^TM^* mice were cultured *in vitro*, and *Prmt5* was deleted by treatment with 1 μM tamoxifen. Bright-field images showed that the clone size of *Prmt5*-deficient SSCs was significantly smaller (Figures 4Gc,d) than that of SSCs treated with ethanol (Figures 4Ga,b). In addition, the MTT assay results also showed that knockout of *Prmt5* resulted in a significant decrease in cell proliferation (Figure 4H). The flow cytometry results showed that the loss of *Prmt5* resulted in G0/G1 phase arrest with a concomitant decrease in S phase (Figures 4I,J). These results indicate that deletion of *Prmt5* results in cell cycle arrest of SSCs cultured *in vitro*.

### *Prmt5-*Deficient Spermatogonial Stem Cells Exhibited Differentially Expressed Genes

**FIGURE 5:**
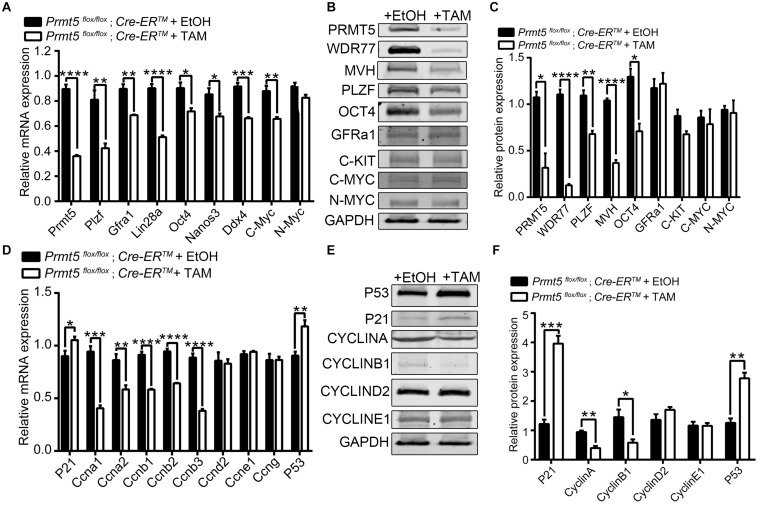
Differentially expressed genes in *Prmt5-*deficient spermatogonial stem cells. The expression of genes related to self-renewal and proliferation was analyzed by real-time PCR **(A)** and western blotting **(B)** in *Prmt5^*flox/flox*^;Cre-ER^TM^* SSCs treated with ethanol or 1 μM tamoxifen. The quantitative results of western blot analysis **(C)**. The expression of cell cycle-associated genes was analyzed by real-time PCR **(D)** and western blot **(E)**. The quantitative results of western blot analysis **(F)**. GAPDH was used as a loading control, and the protein values were normalized. Error bars represent the SEM of triplicate results. *P < 0.05, **P < 0.005, ***P < 0.0005, ****P < 0.0005 indicates a significant difference (*t*-test).

The differentially expressed genes associated with stemness maintenance, proliferation and the cell cycle in both control and *Prmt5*-deficient SSCs were examined by real-time PCR and western blot analysis. *Plzf*, *Oct4*, *Nanos3*, and *Gfra1* are well-characterized pluripotent factors that are important for the maintenance of SSC stemness (Oatley and Brinster, 2008; Chen et al., 2017; Mäkelä and Hobbs, 2019). As shown in Figure 5, the expression of *Prmt5* was dramatically reduced in *Prmt5^*flox*/flox^;Cre-ER^TM^* SSCs treated with 1 μM tamoxifen compared to those treated with ethanol. Both the mRNA and protein levels of stemness-related genes, such as *Oct4* and *Plzf*, were significantly decreased in *Prmt5* knockout SSCs. We also observed that the expression of the germ cell marker gene *Mvh* was significantly decreased in *Prmt5*-deficient SSCs. The mRNA level of *c-Myc* was decreased in *Prmt5*-deficient SSCs (Figure 5A), whereas the protein level was not changed (Figures 5B,C). The expression of cell cycle-associated genes was also examined. As shown in Figures 5D–F, the mRNA and protein levels of A-type cyclins (*Ccna1* and *Ccna2*) and B-type cyclins (*Ccnb1*, *Ccnb2*, and *Ccnb3*) were all significantly reduced in *Prmt5*-deficient SSCs. In contrast, the expression of *cyclin-D*, *cyclin-E1*, and *cyclin-G* was not altered after *Prmt5* inactivation. It is worth noting that *p21* and *p53* expression dramatically increased after inactivation *Prmt5* at both the mRNA and protein levels. These results indicate that PRMT5 is important for stemness maintenance and the proliferation of spermatogonial stem cells.

### H3K9me2 and H3K27me2 Levels Were Significantly Increased in *Prmt5-*Deficient SSCs

**FIGURE 6:**
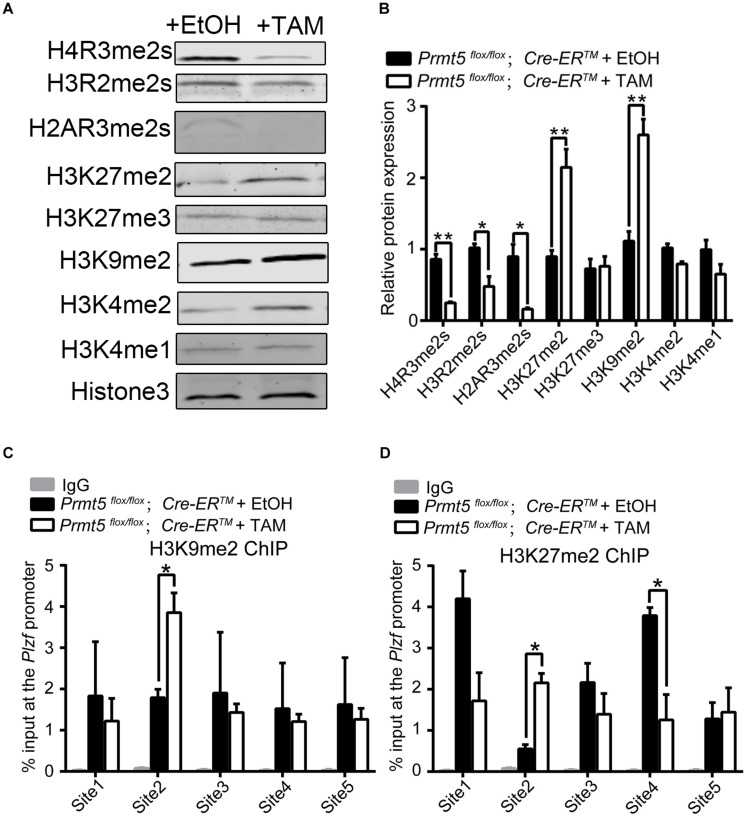
H3K9me2 and H3K27me2 levels are significantly increased in *Prmt5-*deficient spermatogonial stem cells. **(A)** The level of histone modification in control and *Prmt5*-deficient spermatogonial stem cells was examined by western blot. **(B)** Quantitative results of western blot analysis. ChIP assays were performed to analyze the enrichment of H3K27me2 **(C)** and H3K9me2 **(D)** modifications in the promoter regions (TSS-1 kb upstream of TSS) of the *Plzf* (site 1∼ site 5) gene in control and *Prmt5*-deficient SSCs. The relative enrichment of the *Plzf* promoter region was examined by real-time PCR using sequence-specific primer sets. IgG was used as a negative control. Site 1: −114∼−284 bp, Site 2: −175∼−347 bp, Site 3: −381∼−552 bp, Site 4: −573∼−743 bp, Site 5: −755∼−948 bp. Quantitative data are presented as the enrichment of the ChIP to the input DNA. TSS, transcription start site. Error bars present SEM of three ChIP experiments. *P < 0.05, **P < 0.005 indicates a significant difference (*t*-test).

PRMT5 has been shown to regulate the expression of target genes suchas *Blimp1* (Ancelin et al., 2006), *c-Myc* (Liu M. et al., 2020), *p21* (Zhang et al., 2015), and androgen receptor (Deng et al., 2017) via symmetric dimethylation of arginine residues of histones H4 (H4R3), H3 (H3R2 and H3R8), and H2A (H2AR3). The western blot results showed that loss of *Prmt5* led to a dramatic decrease in H4R3me2s, H3R2me2s, and H2AR3me2s levels. Interestingly, the levels of the repressive histone lysine modifications H3K9me2 and H3K27me2 were significantly increased (Figures 6A,B). Previous studies have reported that histone H3 lysine modifications, especially the methylation of H3K9 and H3K27, play important roles in the prospermatogonia to spermatogonia transition and in the development of SPCs/SSCs (Mu et al., 2014; Tseng et al., 2015; Kuroki et al., 2020). To test whether the downregulation of PLZF in *Prmt5*-deficient SSCs is due to a decrease in H4R3me2s, H3R2me2s, and H2AR3me2s levels or an increase in H3K9me2 and H3K27me2, ChIP assays were performed to analyze the enrichment of H4R3me2s, H3R2me2s, H2AR3me2s, H3K9me2, and H3K27me2 at the promoter region (TSS-1 kb upstream of TSS) of the *Plzf* gene. The ChIP-qPCR results showed that H4R3me2s, H3R2me2s, and H2AR3me2s levels remained unchanged (data not shown), whereas those of H3K27me2 and H3K9me2 were significantly increased at the proximal promoter region (Site 2: −175∼−347 bp) of *Plzf* in *Prmt5*-deficient SSCs (Figures 6C,D). These results indicate that the downregulation of *Plzf* in *Prmt5*-deficient SSCs is not directly regulated by *Prmt5* via histone arginine methylation, which is most likely caused by increased H3K27me2 and H3K9me2 levels.

### Expression of Lysine Demethylases of H3K9me2 and H3K27me2 Was Regulated by PRMT5 via Histone Arginine Modifications

**FIGURE 7:**
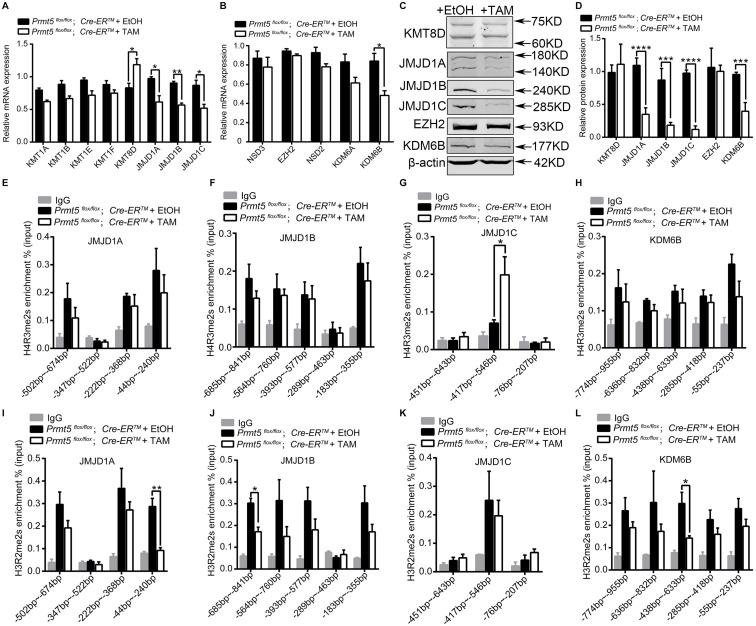
The expression of lysine demethylases of H3K9me2 and H3K27me2 is regulated by PRMT5 via histone arginine modifications. The differential expression of lysine methylases and demethylases of H3K9me2 and H3K27me2 in *Prmt5*-deficient SSCs was analyzed by real-time PCR **(A,B)** and western blot **(C,D)**. β-Actin was used as a loading control, and the protein values were normalized. ChIP-qPCR assays were performed to examine the enrichment of H4R3me2s or H3R2me2s modifications at the upstream regions of JMJD1A **(E,I)**, JMJD1B **(F,J)**, JMJD1C **(G,K)**, and KDM6B **(H,L)** in *Prmt5*-deficient SSCs. The immunoprecipitated DNA fragments were analyzed by real-time PCR with sequence-specific primer sets. IgG was used as a negative control. Quantitative data are presented as the enrichment of the ChIP to the input DNA. Error bars represent the SEM of triplicate results. *P < 0.05, **P < 0.005, ***P < 0.0005, ****P < 0.00005 indicates a significant difference (*t*-test).

To assess the underlying mechanisms that cause the increase in H3K9me2 and H3K27me2 in *Prmt5*-deficient SSCs, we analyzed the expression of lysine methylases and demethylases for H3K9me2 and H3K27me2 by real-time PCR and western blot analysis. As shown in Figure 7, the mRNA level of lysine demethylase for H3K9me2 (JMJD1A, JMJD1B, and JMJD1C) was significantly decreased after *Prmt5* inactivation, whereas the expression of lysine methylases for H3K9me2 (KMT1A, KMT1B, KMT1E, and KMT1F) remained unchanged. The mRNA levels of lysine methylases for H3K27me2 (NSD3, EZH2, and NSD2) were not increased in *Prmt5*-deficient SSCs, while the expression of demethylase for H3K27me2 (KDM6B) was significantly deceased (Figures 7A,B). The western blot results further demonstrated that the expression of the demethylases for H3K9me2 or H3K27me2 was dramatically reduced in *Prmt5*-deficient SSCs (Figures 7C,D). These results suggest that the increase in H3K9me2 and H3K27me2 in *Prmt5*-deficient SSCs is most likely due to the downregulation of demethylases.

To further examine whether the downregulation of lysine demethylase for H3K9me2 (JMJD1A, JMJD1B, and JMJD1C) and H3K27me2 (KDM6B) is caused by changes in histone arginine methylation in *Prmt5*-deficient SSCs, we analyzed the level of H4R3me2s or H3R2me2s at the promoter regions (TSS-1 kb upstream of TSS) of JMJD1A, JMJD1B, JMJD1C, and KDM6B. The ChIP-qPCR results showed that inactivation of *Prmt5* led to a significant increase in the repressive histone modification H4R3me2s at the promoter region (−417 ∼−546 bp) of JMJD1C (Figure 7G), whereas the occupancy of H4R3me2s at the upstream promoters of JMJD1A, JMJD1B, KDM6B was not changed (Figures 7E,F,H). Moreover, the occupancy of permissive histone modification H3R2me2s at the upstream regions (TSS-1 kb upstream of TSS) of the JMJD1A, JMJD1B, and KDM6B genes was significantly reduced in *Prmt5*-deficient SSCs (Figures 7I,J,L). The level of H3R2me2s at the proximal promoter region of JMJD1C was not decreased (Figure 7K). These results suggest that the expression of histone lysine demethylases for H3K9me2 and H3K27me2 is regulated by PRMT5 via histone arginine modifications.

## Discussion

As an epigenetic modifier, PRMT5 has been demonstrated to play important roles in PGC development, and inactivation of this gene caused loss of germ cells during the embryonic stage (Kim et al., 2014; Li et al., 2015; Wang et al., 2015b). The results of our previous study revealed that the postnatal knockout of *Prmt5* in male germ cells using *Stra8-Cre* led to defects in meiosis and male infertility (Wang et al., 2015c). However, the functions of PRMT5 in SSC development have not been previously investigated. In the present study, we demonstrated that PRMT5 is essential for the survival and maintenance of SSCs. The inactivation of *Prmt5* resulted in cell cycle arrest and progressive loss of SSCs at 3 weeks of age.

PLZF has been reported to play important roles in the regulation of diverse cellular processes, including stemness maintenance, differentiation, cell cycle, proliferation and apoptosis (Suliman et al., 2012; Liu et al., 2016). PLZF is also highly expressed in spermatogonial stem cells and is considered a marker gene for undifferentiated spermatogonial stem cells. The deletion of *Plzf* was shown to lead to a progressive loss of spermatogonial stem cells in a mouse model (Buaas et al., 2004; Costoya et al., 2004). Moreover, *Plzf* is considered a cell cycle regulator, and PLZF overexpression in hematopoietic stem cells or the hematopoietic cell line 32Dcl3 was observed to block cells in G1/S phase, resulting in defects in cell growth and differentiation with an increase in apoptosis (Shaknovich et al., 1998; Yeyati et al., 1999; Vincent-Fabert et al., 2016). In the present study, we showed that the expression was dramatically reduced after the loss of *Prmt5.* Based on these results, we speculated that the defect in SSC development in *Prmt5*-deficient mice is most likely due to the downregulation of PLZF.

As a protein arginine methyltransferase, PRMT5 catalyzes MMA or sDMA in histones and non-histone substrates (Rho et al., 2001; Bedford, 2007). Interestingly, we observed that the level of global histone lysine modifications (H3K9me2 and H3K27me2) was significantly increased in *Prmt5-*deficient SSCs. PRMT5-induced H4R3 methylation has been reported to regulate lysine methylation modification (H3K27) via recruitment of Polycomb protein in a Pax2/Grg4-dependent manner (Patel et al., 2012). In hematopoietic cells, *Prmt5* depletion resulted in the upregulation of global H3K27 dimethylation and trimethylation (Liu F. et al., 2020). In the present study, the global level of H3K27me2 was significantly increased, whereas the level of H3K27me3 was not increased in *Prmt5*-deficient SSCs (Figures 6A,B). Moreover, unlike in a previous study (Tae et al., 2011; Patel et al., 2012; Li et al., 2018), the expression of methylases for H3K9me2 and H3K27me2 was not increased in *Prmt5*-deficient SSCs. However, the expression of demethylases (JMJD1A, JMJD1B, JMJD1C, and KDM6B) was significantly reduced at both the protein and mRNA levels, indicating that the increase in H3K9me2 and H3K27me2 was most likely due to the downregulation of histone H3 lysine demethylase expression. These results suggested that the expression of target genes regulated by PRMT5 is cell context dependent.

The functions of histone lysine methylation in SSCs development have been previously reported. The histone lysine demethylase JMJD1 isozymes targeting H3K9me2 play essential roles in the development of SSCs and spermatogenesis. The depletion of both JMJD1A and JMJD1B results in defects in the prospermatogonia to spermatogonia transition and causes abnormal spermatogenesis (Kuroki et al., 2020). The loss of JMJD1C also results in a progressive reduction of SSCs/SPCs and male infertility (Kuroki et al., 2013). The histone H3K27 demethylase KDM6B is involved in regulating the fragmentation of spermatogonial cysts, but the differentiation of SSCs is not affected (Iwamori et al., 2013). EED is a core subunit of Polycomb-repressive complex (PRC2), which is responsible for catalyzing H3K27me2/H3K27me3, and deletion of EED by *Mvh-Cre* leads to defects in SSC maintenance (Mu et al., 2014). Therefore, appropriate epigenetic modification is crucial for the maintenance of the SSC pool and the support of long-term sperm production. In the present study, we concluded that aberrant histone H3 lysine methylation leads to the downregulation of PLZF, which in turn causes defects in SSC maintenance. However, other unknown mechanisms that are regulated by histone modification are probably also involved in this process.

H4R3me2s is a repressive histone arginine modification that is catalyzed by PRMT5 (Zhao et al., 2009; Xu et al., 2010; Deng et al., 2017; Li et al., 2018; Liu M. et al., 2020). The global level of H4R3me2s was dramatically reduced in *Prmt5*-deficient SSCs, consistent with the results of previous studies (Zhao et al., 2009; Wang et al., 2015c; Zhu et al., 2019). Surprisingly, H4R3me2s was enriched at the promoter region of the JMJD1C gene in *Prmt5*-deficient SSCs. These results suggest that H4R3me2s is probably also catalyzed by other arginine methyltransferases and that the increase in H4R3me2s at the promoter region of the JMJD1C gene is probably not directly regulated by PRMT5. H3R2me2s is a permissive histone arginine modification that is also catalyzed by PRMT5 (Kirmizis et al., 2007; Migliori et al., 2012; Yuan et al., 2012; Chiang et al., 2017; Yang et al., 2020). The global level of H3R2me2s was dramatically reduced in *Prmt5*-deficient SSCs, and the levels of H3R2me2s at the promoter regions of JMJD1A, JMJD1B, and KDM6B were all significantly decreased. Based on these results, we concluded that the change in histone arginine methylation in *Prmt5*-deficient SSCs causes the downregulation of histone lysine demethylases. The downregulation of histone lysine demethylase expression causes an increase in H3K9me2 and H3K27me2.

In the present study, we also observed that meiosis was completely blocked in *Prmt5*-deficient germ cells, and no SYCP3 signal was detected in *Prmt5^Δ/flox^;Mvh-Cre* mice at P10. The results of our previous study revealed that deletion of *Prmt5* in male germ cells using *Stra8-Cre* (∼P3) results in aberrant meiotic progression. However, the expression of meiosis-associated genes, such as *Stra8*, *Sycp3*, *Dmc1*, and γ*H2AX*, was not affected (Wang et al., 2015c). Although meiosis was blocked in *Prmt5*-deficient germ cells, we could not conclude that PRMT5 is required for meiosis initiation. The defect of meiosis is probably a consequence of cell cycle arrest of *Prmt5*-deficient SSCs, and the underlying mechanism needs further investigation.

**FIGURE 8:**
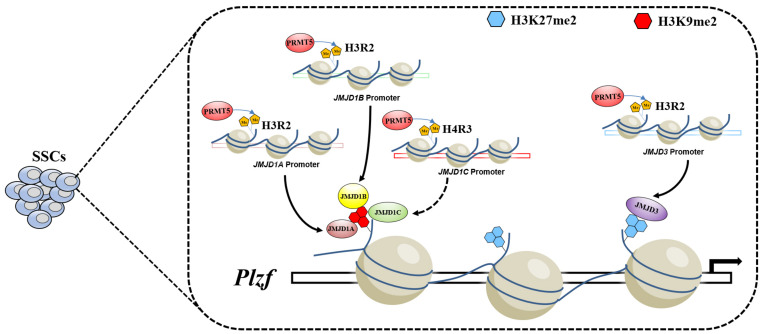
Model of PRMT5 regulating *Plzf* expression via histone modifications in SSCs. In SSCs, PRMT5 promotes the expression of demethylases for H3K9me2 and H3K27me2 by catalyzing permissive histone H3R2me2s. The high expression of the corresponding lysine demethylases leads to low levels of H3K9me2 and H3K27me2 at the upstream promoter of *Plzf*, which in turn leads to the upregulation of the *Plzf* gene.

Taken together, the results of the present study reveal that PRMT5 is involved in regulating the development of spermatogonia and that deletion of *Prmt5* results in depletion of the spermatogonial stem cells pool. We also demonstrated that loss of *Prmt5* caused downregulation of demethylases for H3K9me2 and H3K27me2, which in turn led to an increase in H3K9me2 and H3K27me2 and downregulation of the *Plzf* gene (Figure 8). Our results demonstrated that the crosstalk between histone arginine methylations and histone lysine methylation plays an important role in regulating SSCs development.

## Materials and Methods

### Mice

All mice experiments were carried out in accordance with institutional animal care and the use committee regulations of Institute of Zoology, CAS. All mice were maintained in a C57BL/6;129/SvEv mixed background. *Prmt5^*flox*^* mice were obtained from the European Conditional Mouse Mutagenesis Program (EUCOMM; Prmt5^*tm*2a(EUCOMM)Wtsi^) (Bezzi et al., 2013), *Prmt5^+/Δ^* mice were obtained by crossing with *ZP3-Cre* mice. *Prmt5^Δ/flox^;Mvh-Cre* mice were obtained by crossing *Prmt5^+/Δ^;Mvh-Cre* males with *Prmt5^*flox/flox*^* females. Genotyping was performed by PCR as described previously using DNA isolated from tail tips (Gao et al., 2006; Bezzi et al., 2013; Gao et al., 2014).

### Tissue Collection and Histological Analysis

Testes were dissected from *Prmt5^+/Δ^;Mvh-Cre* and control mice immediately after euthanasia, fixed in 4% paraformaldehyde for up to 24 h, stored in 70% ethanol, and embedded in paraffin. Five-micrometer-thick sections were cut and mounted on glass slides. Then, the tissue sections were processed for immunohistochemistry and immunofluorescence.

### Immunohistochemistry (IHC), Immunofluorescence (IF), and TUNEL Assay

IHC and IF procedures were performed as described previously (Chen et al., 2019; Qin et al., 2019). Antibodies were diluted as follows: MVH (1:500, Abcam, ab13840), PLZF (1:100, R&D, AF2944), PRMT5 (1:200, Millipore, 07-405), SOX9 (1:500, Millipore, AB5535), STRA8 (1:200, Abcam, ab49405), SYCP3 (1:200, Abcam, ab15093), Ki67 (1:400, Abcam, ab15580), and PH3 (1:400, Millipore, 2605439). After staining, the sections were examined with a Nikon microscopy, and images were captured with a Nikon DS-Ri1 CCD camera. The IF sections were examined using a confocal laser scanning microscope (Carl Zeiss Inc., Thornwood, NY, United States). TUNEL assay was performed using the Dead-End Fluorometric TUNEL System (Promega, G3250).

### Spermatogonial Stem Cells *in vitro* Culture

Establishment and maintenance of SSCs were performed as previously described (Wang et al., 2015a; Chen et al., 2017). In brief, seminiferous tubules from *Prmt5^*flox/flox*^;Cre-ER^TM^* mice at 5–7 days postpartum (dpp) were digested with collagenase IV and DNase I for 5 min into small fragments and then centrifuged at 400 rpm for 2 min. The seminiferous fragments were suspended in mouse embryo fibroblast (MEF) medium containing 10% FBS (fetal bovine serum). Twenty-four hours later, the SSCs were collected and then transferred to mitomycin C-treated MEF feeder cells. The specific medium for SSCs contained GDNF and FGF2 for later culture. *Prmt5^*flox/flox*^;Cre-ER^TM^* SSCs treated with ethanol or 1 μM tamoxifen were harvested for western blot, real-time PCR or MTT assay.

### Western Blotting and Antibodies

Western blotting procedures were performed as described previously (Zhou et al., 2018). Tissues and cells were lysed in cold RIPA buffer, supplemented with 1 mM phenylmethylsulfonyl fluoride and protease inhibitor cocktail (Roche, Indianapolis, IN, United States). The protein lysates were subjected to SDS-PAGE, transferred onto a nitrocellulose membrane and probed with the primary antibodies. The images were captured with the ODYSSEY Sa Infrared Imaging System (LI-COR Biosciences, Lincoln, NE, United States). Primary antibodies used were shown in Supplementary Table 1.

### Nucleic Acid Isolation and Quantitative Real-Time PCR

For Real-Time PCR, RNA was isolated from cultured SSCs using EASYspin Plus Tissue/Cells RNA Rapid Extraction kit following manufacturer’s instructions. The relative expression level was calculated using the formula 2^–ΔΔ*CT*^. *Gapdh* was used as an internal control for quantification. The primers used were listed in Supplementary Table 2.

### Cell Proliferation Assay

The relative number and viability of SSCs were evaluated by MTT assays. In brief, SSCs seeded in 24-well plates were washed with PBS twice and incubated with MTT solution (0.5 mg/ml) for at least 4 h at 37°C in a CO_2_ incubator. Then, the medium containing MTT was removed, and 750 μl DMSO was added. After an incubation for 10 min on a shaking table at 75 rpm/min, the OD value at 490 nm was measured.

### ChIP-qPCR Assay

ChIP assays were conducted using a SimpleChIP^®^ Plus Sonication Chromatin IP Kit (# 56383) following the manufacturer’s instructions. For ChIP-qPCR, the input genomic DNA or immunoprecipitated DNA was used as a template. Quantitative real-time PCR was performed according to the manufacturer’s instructions. The input DNA was used as a normalization control. The primers used are listed in Supplementary Table 2.

### Statistics Analysis

Experiments were repeated at least three times. GraphPad Prism7 software was used for analysis of *P*-values based on three to six independent experiments in PCR reactions or western blotting assay. Multiple *t*-test-one per row was used to compare the differences between two groups. *P* < 0.05 or *P* < 0.01 was considered statistically significant.

## Data Availability Statement

The original contributions presented in the study are included in the article/Supplementary Material, further inquiries can be directed to the corresponding author/s.

## Ethics Statement

The animal study was reviewed and approved by the Institutional Animal Care and Use Committee (IACUC) of the Institute of Zoology, CAS (AEI-09-02-2014).

## Author Contributions

FD and MC (co-first author): formal analysis and investigation. FG: funding acquisition. FD, MC (co-first author), MC (third author), LJ, ZS, LM, CH, and XG: methodology. FG: project administration. MC (co-first author) and LM: resources. FD: writing—original draft. FD and FG: writing—review and editing.

## Conflict of Interest

The authors declare that the research was conducted in the absence of any commercial or financial relationships that could be construed as a potential conflict of interest.
