# Supplementary material for: PRMT5 Is Involved in Spermatogonial Stem Cells Maintenance by Regulating Plzf Expression via Modulation of Lysine Histone Modifications
Source: Front Cell Dev Biol. 2021 May 21;9:673258. doi: 10.3389/fcell.2021.673258 (PMC8185031; doi:10.3389/fcell.2021.673258)
Supplement: Supplementary file 3 [file Data_Sheet_1.PDF]

Supplementary Figures and Figure legends

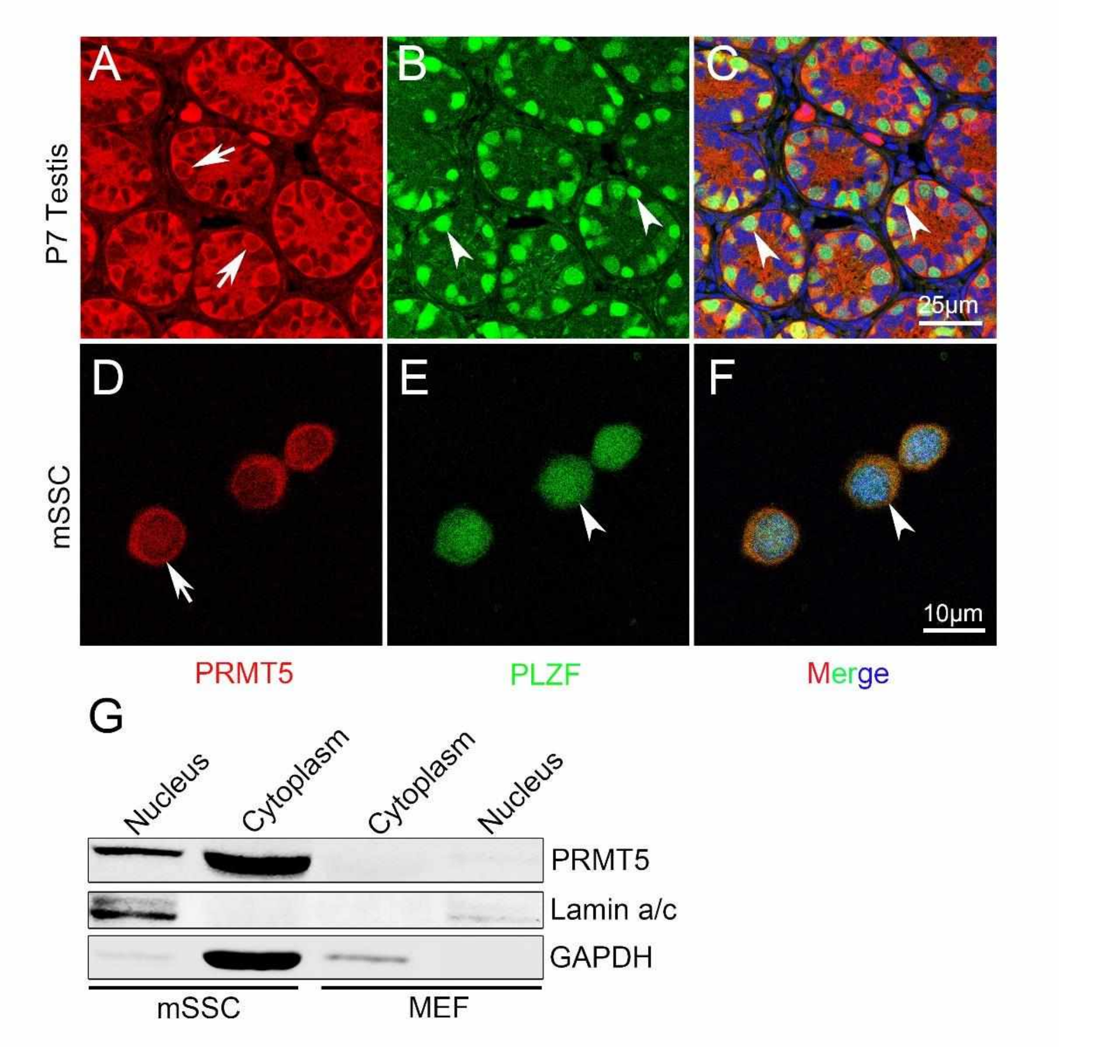

Supplementary Figure 1. PRMT5 was abundantly expressed in spermatogonial stem cells. The expression of PRMT5 in P7 testis **(A-C)** and in vitro cultured spermatogonial stem cells **(D-F)** was examined by Immunofluorescence. PRMT5 was mainly detected in the cytoplasm of germ cells **(A, white arrows)** and germ cells were labeled with anti-PLZF antibody **(B, C, green, white arrowheads)**. PRMT5 was expressed in both nucleus and cytoplasm of in vitro cultured spermatogonial stem cells **(D, white arrows)**. PLZF was expressed in the cultured spermatogonial stem cells **(F, green, white arrowheads)**. PRMT5 protein was detected in both nucleus and cytoplasm of in vitro cultured spermatogonia stem cells by western blot assay. Lamin a/c and GAPDH were served as loading control of nucleus and cytoplasm extracts **(G)**.

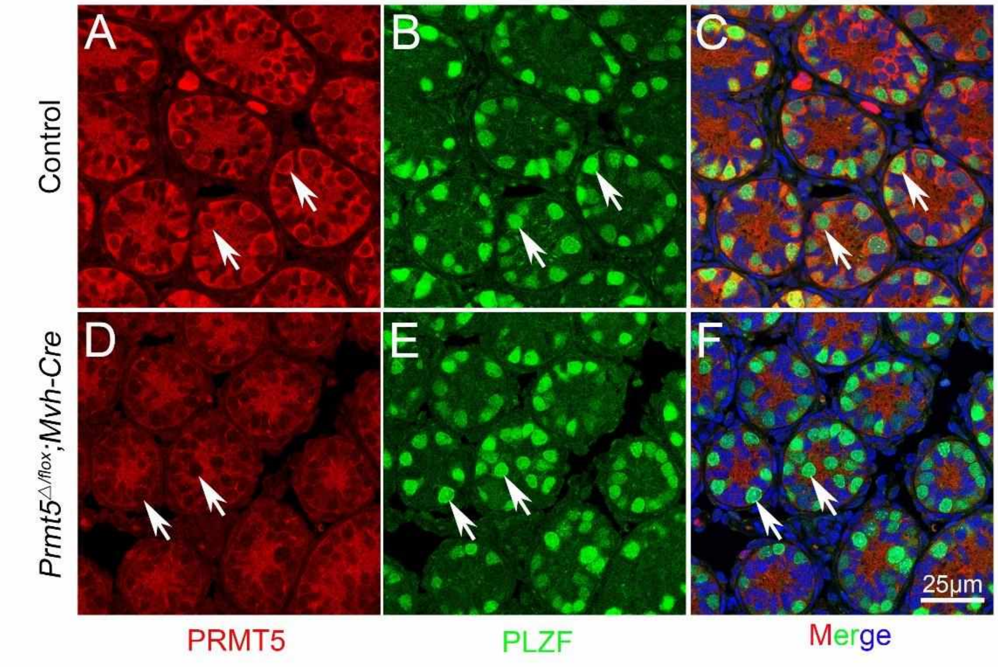

Supplementary Figure 2. PRMT5 was not detected in spermatogonia stem cells of *Prmt5△/flox;Mvh-Cre* mice The expression of PRMT5 in testis of control **(A-C)** and *Prmt5△/flox;Mvh-Cre* mice **(D-F)** at P7 was examined by immunofluorescence. The germ cells were labeled with anti-PLZF antibody **(B, C, D, F, green, white arrows)**. PRMT5 was abundantly expressed in the germ cells of control testes **(A, C, red, white arrows)**, but not in the germ cells of *Prmt5△flox;Mvh-Cre* mice **(D, F, red, white arrows)**.

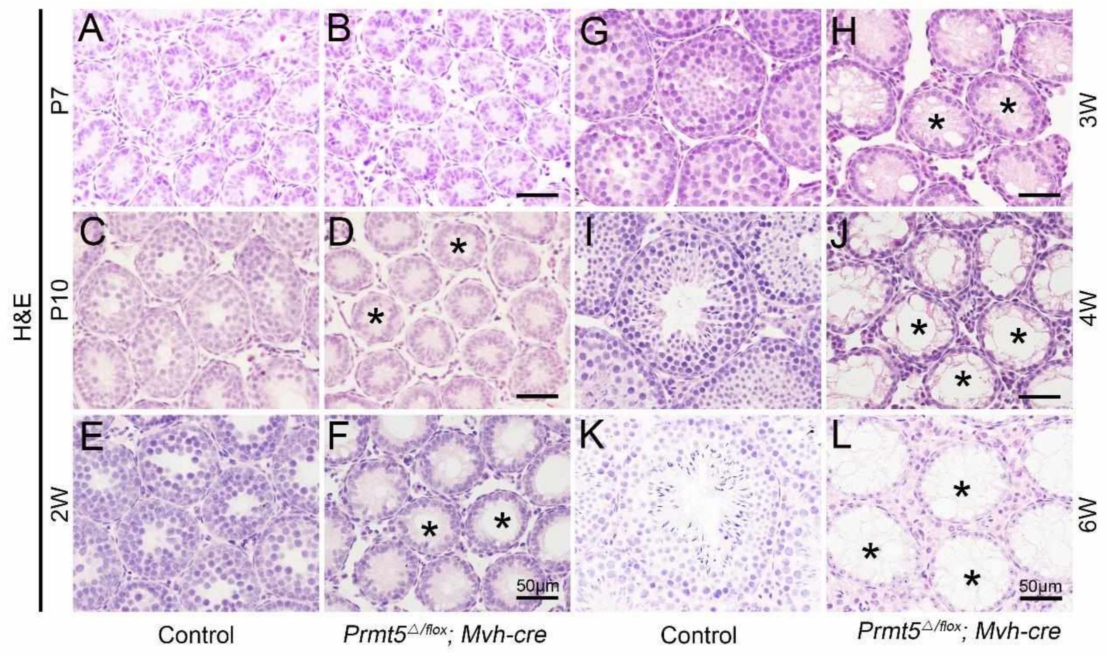

Supplementary Figure 3. The defect of germ cell development was first observed in *Prmt5*$^{\Delta/flox}$*;Mvh-Cre* mice at P10. Testes from control and *Prmt5*$^{\Delta/flox}$*;Mvh-Cre* at P7, P10, 2W, 3W, 4W and 6W **(A-L)** were stained with H&E. The testes from *Prmt5*$^{\Delta/flox}$*; Mvh-Cre* mice **(B)** was grossly normal at P7 compared to that of control testes **(A)**. Aberrant seminiferous tubules were first noted in *Prmt5*$^{\Delta/flox}$*;Mvh-Cre* testes **(D, asterisks)** at P10. Empty tubules were observed in *Prmt5*-deficient testes at 2W, 3W 4W, and 6W **(F, H, J, L, asterisks)**.

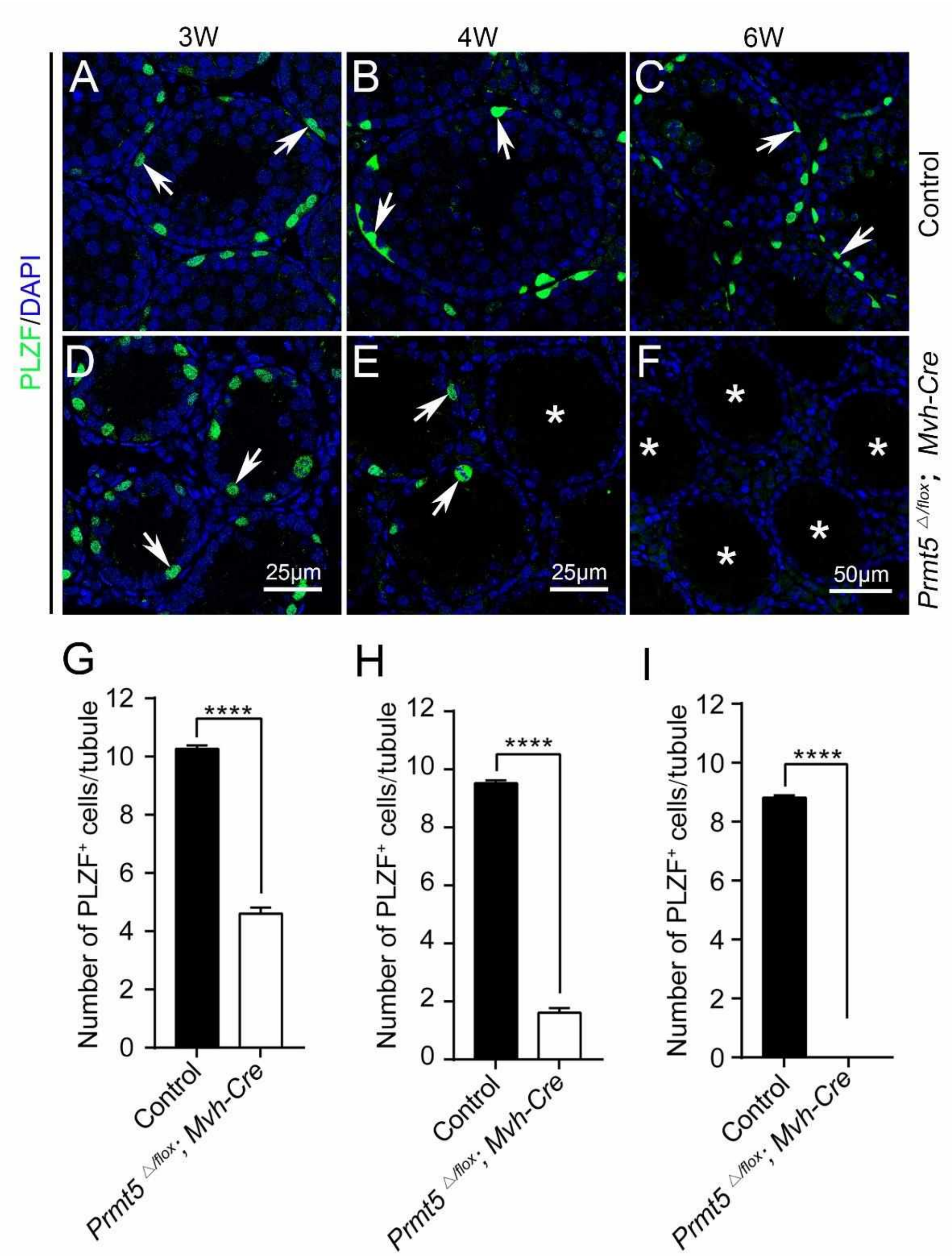

Supplementary Figure 4. PLZF-positive spermatogonia were gradually lost in *Prmt5$^{\Delta/flox}$;Mvh-Cre* mice. PLZF-positive spermatogonia (green) were observed in the seminiferous tubules of control mice at 3 weeks **(A, white arrows)**, 4 weeks **(B, white arrows)**, and 6 weeks **(C, white arrows)**. PLZF-positive spermatogonia (green) were also observed in the seminiferous tubules of *Prmt5$^{\Delta/flox}$;Mvh-Cre* mice at 3 weeks **(D, white arrows)** and 4 weeks **(E, white arrows)**, but no germ cell was observed at 6 weeks **(F, asterisks)**. Quantitative results showed that the number of PLZF-positive germ cells was dramatically reduced in *Prmt5$^{\Delta/flox}$;Mvh-Cre* mice at 3 weeks **(G)** and 4 weeks **(H)**, and no germ cell was counted at 6 weeks **(I)**. Error bars represent SEM of triplicates. (****) $P < 0.00001$ indicates statistically significant changes (t-test).
